# Speech pattern disorders in verbally fluent individuals with autism spectrum disorder: a machine learning analysis

**DOI:** 10.3389/fninf.2025.1647194

**Published:** 2025-10-24

**Authors:** Chuanbo Hu, Jacob Thrasher, Wenqi Li, Mindi Ruan, Xiangxu Yu, Lynn K. Paul, Shuo Wang, Xin Li

**Affiliations:** ^1^Department of Computer Science, University at Albany, Albany, NY, United States; ^2^Lane Department of Computer Science and Electrical Engineering, West Virginia University, Morgantown, WV, United States; ^3^Department of Radiology, Washington University in St. Louis, St. Louis, MO, United States; ^4^Humanities and Social Sciences, California Institute of Technology, Pasadena, CA, United States

**Keywords:** speech pattern, ASD, machine learning, ADOS, audio, medical dialogues

## Abstract

**Introduction:**

Diagnosing Autism Spectrum Disorder (ASD) in verbally fluent individuals based on speech patterns in examiner-patient dialogues is challenging because speech-related symptoms are often subtle and heterogeneous. This study aimed to identify distinctive speech characteristics associated with ASD by analyzing recorded dialogues from the Autism Diagnostic Observation Schedule (ADOS-2).

**Methods:**

We analyzed examiner-participant dialogues from ADOS-2 Module 4 and extracted 40 speech-related features categorized into intonation, volume, rate, pauses, spectral characteristics, chroma, and duration. These acoustic and prosodic features were processed using advanced speech analysis tools and used to train machine learning models to classify ASD participants into two subgroups: those with and without A2-defined speech pattern abnormalities. Model performance was evaluated using cross-validation and standard classification metrics.

**Results:**

Using all 40 features, the support vector machine (SVM) achieved an F1-score of 84.49%. After removing Mel-Frequency Cepstral Coefficients (MFCC) and Chroma features to focus on prosodic, rhythmic, energy, and selected spectral features aligned with ADOS-2 A2 scores, performance improved, achieving 85.77% accuracy and an F1-score of 86.27%. Spectral spread and spectral centroid emerged as key features in the reduced set, while MFCC 6 and Chroma 4 also contributed significantly in the full feature set.

**Discussion:**

These findings demonstrate that a compact, diverse set of non-MFCC and selected spectral features effectively characterizes speech abnormalities in verbally fluent individuals with ASD. The approach highlights the potential of context-aware, data-driven models to complement clinical assessments and enhance understanding of speech-related manifestations in ASD.

## 1 Introduction

Autism spectrum disorder (ASD) is a developmental condition that presents considerable challenges in social interaction, communication, and behavior (Leekam et al., 2011; Lord et al., 2018, 2020). In the United States, ASD affects approximately 1 in 36 children and 1 in 45 adults, making it a critical public health concern (Maenner, 2020; Dietz et al., 2020). Despite its prevalence, diagnosing ASD is complex, relying heavily on subjective assessments of behavior and the clinical expertise of specialists. These complexities are compounded by differences in diagnostic standards and healthcare availability across regions, resulting in delayed diagnoses and limiting early intervention opportunities for many families (Daniels and Mandell, 2014). This subjectivity can lead to inconsistencies in the accuracy and timing of diagnoses across various regions and populations.

ASD diagnosis is traditionally conducted through clinical interviews and behavioral observations, often following standardized tools such as the Autism Diagnostic Observation Schedule (ADOS) (Lord et al., 1999). ADOS-2 consists of five modules, each tailored to different age groups and language abilities, ranging from nonverbal toddlers to verbally fluent adults. Module 1 is designed for minimally verbal children, Module 2 for those with some phrase speech, Module 3 for verbally fluent children and adolescents, Module 4 for verbally fluent adults, and the Toddler Module for children under 30 months of age. This structured approach allows clinicians to assess social communication, interaction, and restricted or repetitive behaviors across diverse developmental stages. However, these methods require extensive clinician expertise, leading to potential inconsistencies in diagnosis and accessibility issues in underserved areas (Matson and Kozlowski, 2011; Elsabbagh et al., 2012). ADOS-2 assessments require trained clinicians who can administer structured tasks, score behavioral responses, and interpret results based on standardized criteria. This specialized training is costly and time-intensive, contributing to a shortage of qualified professionals, especially in regions with limited healthcare resources. Moreover, ASD evaluations are often expensive, requiring multiple clinical visits, making it difficult for families in lower-income communities to access timely assessments. As a result, there is increasing interest in technology-driven approaches that can enhance diagnostic consistency and accessibility (Fletcher-Watson and Happé, 2019; Song et al., 2019; Rezaee, 2025).

One promising approach is the use of speech analysis for ASD detection. Speech is a fundamental mode of communication, and research suggests that individuals with ASD often exhibit distinctive speech characteristics, including atypical intonation, altered rhythm, abnormal speech rate, and variations in pitch modulation (Mody and Belliveau, 2013; Pickles et al., 2009; Vogindroukas et al., 2022; Martin and Rouas, 2024). These abnormalities can emerge in development, offering a potential biomarker for ASD diagnosis (Bonneh et al., 2011). Advances in computational speech processing enable precise analysis of these features, paving the way for non-invasive, scalable, and cost-effective diagnostic tools that could complement existing clinical methods.

Recent advancements in machine learning have further expanded the possibilities for ASD diagnosis by enabling automated detection of behavioral and linguistic patterns (Wang et al., 2015; Ruan et al., 2021, 2023; Zhang et al., 2022). For example, machine learning techniques have been applied to digital behavioral phenotyping (Perochon et al., 2023) and automated analysis of gestures and facial expressions from video recordings (Lakkapragada et al., 2022; Krishnappa Babu et al., 2023). Natural language processing (NLP) has also been applied to electronic health records to derive ASD phenotypes (Zhao et al., 2022). Speech features are increasingly recognized as digital biomarkers in clinical decision support (Sariyanidi et al., 2025). Advances in representation learning, such as GANs and self-supervised models, have demonstrated improved ASD speech recognition performance, even in data-limited conditions (Sohn et al., 2025; Al Futaisi et al., 2025). On a different scale, Rajagopalan et al. (2024) showed that robust prediction can be achieved with minimal feature sets across large cohorts, while multi-modal approaches such as facial expression analysis are emerging as valuable complements to speech-based diagnosis (Mahmood et al., 2025). Building on these successes, leveraging ML for speech analysis offers a promising and relatively unexplored direction in ASD diagnosis.

This study targets verbally fluent individuals assessed with ADOS-2 Module 4 and classifies participants with vs. without A2-defined speech abnormalities. Our goal is not to distinguish ASD from non-ASD; rather, we examine how machine learning can characterize speech-related abnormalities within this subgroup and how such models might complement clinical practice. This research focuses on the following key objectives:

**Comprehensive speech feature extraction:** we employed advanced signal processing techniques to extract 40 distinct speech features, grouped into prosodic, rhythmic, spectral, and energy-related categories, to capture subtle ASD-related speech patterns.

**Machine learning-based classification:** we applied machine learning models to classify participants with vs. without ADOS-2 A2-defined speech abnormalities, providing an objective framework for analyzing atypical prosody and rhythm.

**Complementary clinical insight:** Rather than diagnosing ASD *per se*, this study evaluates whether acoustic speech features can support the characterization of speech abnormalities in verbally fluent individuals with ASD, serving as a data-driven complement to traditional clinical assessments.

This study represents a significant methodological advancement in diagnosis of speech abnormalities in ASD by integrating machine learning with detailed speech analysis. The use of a comprehensive set of speech features, combined with sophisticated machine learning techniques, offers a notable improvement over traditional diagnostic methods. This approach holds the potential for more accurate and earlier detection of ASD, which is critical for timely intervention. Ultimately, the research aims to contribute to personalized treatment and management strategies, enhancing outcomes for individuals with ASD and providing a scalable, objective solution for clinical use. This work focuses on autistic individuals assessed with ADOS-2 Module 4 (verbally fluent adolescents and adults); accordingly, findings pertain to this subgroup rather than the autism spectrum as a whole.

## 2 Methods

### 2.1 Caltech audio dataset

#### 2.1.1 Autism Diagnostic Observation Schedule (ADOS)

The Autism Diagnostic Observation Schedule, Second Edition (ADOS-2) (Lord et al., 1999; American Psychiatric Association et al., 2013) is a widely used standardized instrument for diagnosing ASD. Module 4 of ADOS-2 is specifically designed for verbally fluent adolescents and adults, typically aged 16 and older, and differs from other modules intended for younger or non-verbal individuals. This study focuses on the A2 score, which assesses abnormalities in speech patterns, including intonation, volume, rate, and rhythm. Details for each A2 score level are provided in Table 1.

**Table 1:** Speech abnormalities associated with autism (intonation/volume/rhythm/rate).

**Score**	**Description**
0	Appropriately varying intonation, reasonable volume, and normal rate of speech, with regular rhythm coordinated with breathing.
1	Little variation in pitch and tone; rather flat or exaggerated intonation, but not obviously peculiar, OR slightly unusual volume, AND/OR speech that tends to be somewhat unusually slow, fast, or jerky.
2	Speech that is clearly abnormal for ANY of the following reasons: slow and halting; inappropriately rapid; jerky and irregular in rhythm (other than ordinary stutter/stammer), such that there is some interference with intelligibility; odd intonation or inappropriate pitch and stress; markedly flat and toneless ("mechanical"); consistently abnormal volume.
7	Stutter or stammer or other fluency disorder (if odd intonation is also present, code 1 or 2 accordingly).

#### 2.1.2 ADOS interview audio dataset

**Figure 1:**
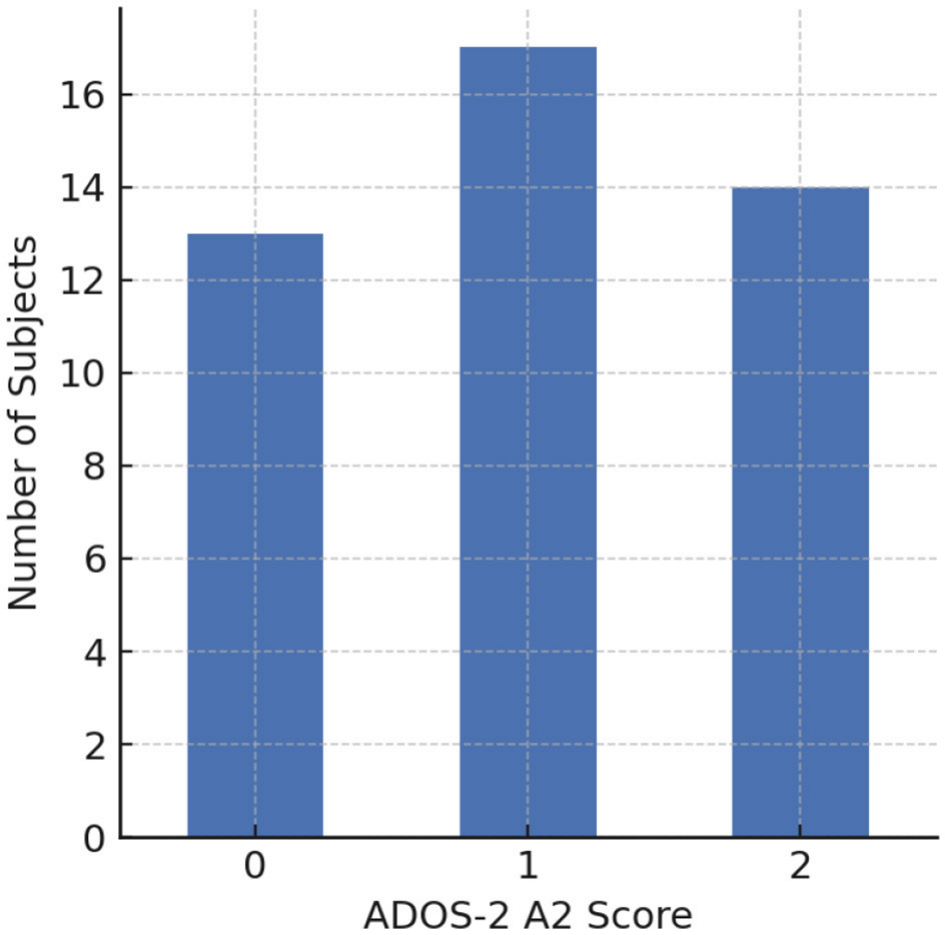
Distribution of ADOS-2 Module 4 A2 scores across subjects (0 = normal intonation, 1 = mildly atypical intonation, 2 = markedly atypical intonation).

The ADOS sessions were conducted sequentially, involving 15 structured scenario tasks designed to elicit responses across a range of communicative and social interactions (see Table 2). These tasks allow clinicians to capture meaningful speech and behavioral data, including intonation and speech rate, for analysis. In this study, the Caltech Audio Dataset (Zhang et al., 2022) includes 33 verbally fluent participants with ASD (26 male, 7 female), aged 16-37 years. The average age of ASD participants was 23.45 ± 4.76 years. Nine of these individuals were assessed twice, approximately six months apart, yielding a total of 42 recording sessions. As shown in Figure 1, 19 participants exhibited speech abnormalities (A2 ≥ 1), while 14 participants received an A2 score of 0. Based on this distribution, the recordings were grouped into ASD with vs. without speech-related abnormalities. To enhance granularity and contextual specificity, each session was further segmented into 15 structured scenario tasks, resulting in 42 × 15 = 630 scenario-level samples, which served as the basic units for subsequent binary classification analyses.

**Table 2:** Overview of SCENARIO TASKS in ADOS-2 module 4 diagnosing process.

**Scenario**	**Name**	**Explanation**
*S*_1_	Construction Task	Involves the participant engaging in a task that requires constructing or assembling a set structure, testing spatial and motor skills, rather than communicative abilities.
*S*_2_	Telling a Story from a Book	Primarily a monologic task where the participant recounts a story from a book, differing from spontaneous dialogic interactions.
*S*_3_	Description of a Picture	Participants describe a picture, testing their ability to interpret visual information and articulate a coherent description.
*S*_4_	Conversation and Reporting	Focuses on the ability to engage in back-and-forth conversation and to report on past events.
*S*_5_	Current Work and School	Discusses participants' current educational and occupational engagements.
*S*_6_	Social Difficulties and Annoyance	Elicits experiences of social challenges and annoyances.
*S*_7_	Emotions	Requires participants to express and identify emotions.
*S*_8_	Demonstration Task	Requires the participant to demonstrate how to use an item or explain a process, which does not involve interactive communication with an examiner.
*S*_9_	Cartoons	Involves interpreting sequences and explaining cartoon strips.
*S*_10_	Break	A pause or intermission in the assessment, involving no communicative or cognitive tasks.
*S*_11_	Daily Living	Covers daily routines and personal care tasks.
*S*_12_	Friends, Relationships, and Marriage	Discusses personal relationships and social norms regarding friendships and marital status.
*S*_13_	Loneliness	Addresses feelings and situations of loneliness and isolation.
*S*_14_	Plans and Hopes	Involves discussing future aspirations and plans.
*S*_15_	Creating a Story	Tests creative storytelling abilities in an unstructured task.

In addition, although the age range (16-37 years) may overlap with vocal maturation for some participants, we did not explicitly control for or model potential pubertal voice changes. Because our feature set includes acoustic descriptors (e.g., spectral measures), such effects cannot be fully ruled out; we therefore acknowledge this as a limitation and a direction for future, age-stratified analyses.

### 2.2 Feature extraction for identification of autism speech disorder

Feature extraction plays a crucial role in the analysis of speech data, especially in understanding complex disorders like ASD. It involves quantifying various aspects of speech that may reveal traits associated with ASD. For this study, a comprehensive set of speech features was extracted from recorded dialogues, grouped based on their relevance to ASD. Prosodic speech features, including the number of syllables, pauses, rate of speech, articulation rate, speaking duration, original duration, balance, and frequency, were extracted using the “Myprosody” tool (Shahab, 2025). This tool integrates multiple speech feature extraction methods, providing a detailed analysis of prosodic elements. Additionally, features such as Mel-Frequency Cepstral Coefficients (MFCCs), spectrograms, and chromagrams were extracted using “pyAudioAnalysis” (Giannakopoulos, 2015), enriching the dataset with diverse audio representations that are essential for analyzing ASD-related speech patterns. These features are described below and summarized in Table 3.

**Table 3:** Detailed categorization of speech features into relevant categories, with explanations and specific feature counts, tailored for comprehensive speech pattern analysis in clinical assessments such as autism.

**No**.	**Category**	**Features**	**Explanation**	**#**
1	Intonation	Frequency	Fundamental frequency, related to the pitch of the voice.	1
		MFCCs	Mel Frequency Cepstral Coefficients, capture timbral aspects that are crucial for intonation.	13
2	Volume	Energy	Measures the signal's loudness.	1
		Entropy of Energy	Indicates variation in loudness within a frame.	1
3	Rhythm	Zero Crossing Rate (ZCR)	Reflects the number of times the waveform crosses zero, related to the frequency of the signal.	1
4	Rate	Rate of Speech	Measures how fast words are spoken.	1
		Number of Syllables	Counts the syllables, indicating speech density and pace.	1
5	Pause	Number of Pauses	Total pauses, reflecting speech interruptions and flow.	1
		Balance	Ratio of speaking to pausing, indicates rhythmic flow.	1
6	Spectral	Spectral Centroid	Center of gravity, affects perceived pitch and sharpness.	1
		Spectral Spread	Measures the width of the spectrum, related to the sharpness of sound.	1
		Spectral Rolloff	The frequency below which 90% of energy lies, indicates the shape.	1
		Spectral Flux	Measures the changes between frames, indicates rhythm changes.	1
		Spectral Entropy	Reflects the entropy of spectral distribution, a complexity measure.	1
7	Chroma	Chroma	A set of 12 coefficients each representing a semitone within an octave, used in harmony analysis.	12
8	Duration	Speaking Duration	measure speaking time (excluding fillers and pause)	1
		Original Duration	measure speaking time (including fillers and pause)	1

Each category of features captures different characteristics of speech that are potentially altered in ASD:

- **Prosody features** such as pitch (fundamental frequency) variations and speech rate are directly related to the emotional and syntactical aspects of speech, which are often atypical in ASD.
- **Energy and Zero Crossing Rate** provide basic information about the speech amplitude and frequency, which are useful for detecting abnormalities in speech loudness and pitch changes.
- **Spectral and Chroma features** reflect the quality of sound and harmony in speech. These features are sophisticated and can detect subtleties in speech that are not apparent through simple auditory observation.
- **MFCCs and their deltas** offer a robust representation of speech based on the human auditory system's perception of the frequency scales, essential for identifying nuanced discrepancies in how individuals with ASD perceive and produce sounds. By analyzing these features using machine learning models, we aim to identify patterns that are indicative of ASD, thereby assisting in the objective and efficient diagnosis of the disorder.

### 2.3 Classification models for diagnosis of speech abnormalities in ASD and analysis

To classify ASD-related speech patterns, we employed six machine learning algorithms, selected based on their effectiveness in speech processing and biomedical signal classification. The classification process follows three major stages:

(1) Model selection based on suitability for structured and unstructured speech features,
(2) Feature selection and optimization to improve performance, and
(3) Model interpretability to analyze which speech features contribute most to classification.

#### Model selection rationale

Each model was selected based on its unique advantages in handling high-dimensional, speech-derived features:

- Support Vector Machine (SVM) (Cortes, 1995): Works well in high-dimensional spaces and can handle non-linear decision boundaries using Radial Basis Function (RBF) kernels.
- Random Forest (RF) (Breiman, 2001): An ensemble learning approach that enhances prediction stability by aggregating multiple decision trees.
- Gradient Boosting (GB) (Friedman, 2001): Sequentially builds trees to correct errors of previous iterations, optimizing for complex non-linear relationships.
- Adaptive Boosting (AdaBoost) (Freund and Schapire, 1997): Assigns higher weights to misclassified samples, improving generalization while being prone to noise sensitivity.
- K-Nearest Neighbors (KNN) (Fix and Hodges, 1951): A distance-based classifier, useful when labels have well-separated clusters in feature space.
- Naïve Bayes (NB) (Rish et al., 2001): A probabilistic model assuming feature independence, known for fast training and robust results in speech applications.

Each model was implemented in Python (Scikit-Learn) and trained using 5-fold cross-validation to assess robustness.

#### Hyperparameter tuning

Hyperparameters were optimized using grid search and random search techniques:

- Grid Search: Exhaustive search of pre-defined parameter sets for SVM, Random Forest, and Boosting models.
- Random Search: Used for KNN and AdaBoost, where sampling over parameter space provides efficient exploration.

Each model's hyperparameter settings are detailed in Table 4.

**Table 4:** Machine learning models and hyperparameter settings for ASD classification.

**Model**	**Hyperparameters**
SVM	C=0.1, Kernel=RBF, Gamma=scale, Tolerance=1e-3, Max Iterations=-1
RF	Trees=100, Max Depth=None, Min Samples Split=10, Min Samples Leaf=5, Bootstrap=True
GB	Learning Rate=0.1, Trees=100, Max Depth=3, Min Samples Split=5, Subsample=0.8
AdaBoost	Estimators=50, Learning Rate=1.0, Base Estimator=Decision Stump, Algorithm=SAMME.R
KNN	K=5, Distance=Euclidean, Weights=Uniform, Algorithm=Auto, Leaf Size=30
NB	Distribution=Gaussian, Variance Smoothing=1e-9

The performance of each model was assessed using multiple metrics, including accuracy, precision, recall, and F1-score, calculated through cross-validation across the dataset.

In addition, we employed 5-fold GroupKFold cross-validation to evaluate model performance, ensuring that recordings from the same participant were not split across folds. This choice was made to balance bias and variance in model evaluation, given the limited dataset size.

### 2.4 Feature importance evaluation

To enhance transparency in ASD classification, we applied several interpretability techniques to analyze feature contributions. Shapley Additive Explanations (SHAP) (Lundberg and Lee, 2017) was employed to estimate the impact of each speech feature on model predictions. SHAP values were computed for all samples, allowing us to examine both individual and global feature influences. SHAP was chosen because it provides consistent, theoretically grounded attributions that are model-agnostic, making it especially suitable for comparing feature relevance across diverse classifiers (e.g., SVM, Random Forest, Gradient Boosting). Alternative methods such as LIME, permutation importance, or partial dependence plots (PDP) were considered; however, SHAP was prioritized due to its ability to capture both local and global interpretability in a unified framework. We acknowledge that SHAP is computationally more expensive than these alternatives, and this aspect is discussed further in the Limitations section. This approach provided insight into how changes in speech characteristics affect classification probability, facilitating a better understanding of model decisions.

For tree-based models such as Random Forest and Gradient Boosting, feature importance was derived using the Mean Decrease in Impurity (MDI) metric. This method ranks features based on their contribution to reducing uncertainty in classification. Additionally, we applied permutation importance to models that do not natively provide feature rankings, such as SVM and KNN. By randomly shuffling each feature and measuring its effect on model performance, we identified the most influential features for ASD classification.

Given that ADOS-2 Module 4 consists of 15 structured tasks, we conducted a scenario-specific feature analysis to investigate whether feature importance varies across different conversational contexts. This analysis involved computing SHAP values separately for each task, allowing us to assess how models rely on specific speech features under varying conditions.

To further interpret model decisions, we incorporated visualization techniques, including SHAP summary plots, feature importance rankings, and scenario-wise importance heatmaps. These visual tools help illustrate patterns in speech-related features and aid in understanding how classification decisions are made. By integrating multiple interpretability methods, we aimed to ensure that our models remain transparent and suitable for potential clinical applications.

The combination of SHAP analysis, feature ranking, and visualization techniques allows for a comprehensive assessment of model behavior. These interpretability methods provide essential insights for refining ASD classification models, validating the consistency of learned patterns, and supporting future improvements in automated diagnostic tools.

## 3 Results

### 3.1 Experimental setup

To evaluate model performance, we applied a supervised classification framework using the extracted speech features. All experiments were conducted in Python (Scikit-learn) with 5-fold cross-validation to ensure robustness and reduce overfitting. Models were trained and tested on both feature sets described in Section 2.3 (the full 40-feature set and the reduced 15-feature set). We assessed diagnostic performance using four standard classification metrics:

- **Accuracy**: The proportion of correctly classified samples out of all samples.
- **Precision**: The proportion of predicted positive cases that are true positives, measuring the reliability of positive predictions.
- **Recall (Sensitivity)**: The proportion of true positive cases correctly identified, reflecting the ability to capture actual ASD cases.
- **F1-score**: The harmonic mean of precision and recall, balancing the trade-off between false positives and false negatives.

Formally, given true positives (TP), false positives (FP), false negatives (FN), and true negatives (TN):


$$\begin{array}{l} \text{Accuracy}=\frac{TP+TN}{TP+TN+FP+FN}\\ \text{Precision}=\frac{TP}{TP+FP},\text{ Recall}=\frac{TP}{TP+FN},\\ \text{ F1 }-\text{ score}=2\times \frac{\text{Precision}\times \text{Recall}}{\text{Precision}+\text{Recall}} \end{array}$$

These metrics are widely used in medical classification tasks and provide complementary perspectives on diagnostic reliability. Accuracy summarizes overall performance, precision emphasizes avoiding false positives, recall emphasizes capturing true cases, and the F1-score balances both aspects.

### 3.2 Analysis of speech pattern features

**Figure 2:**
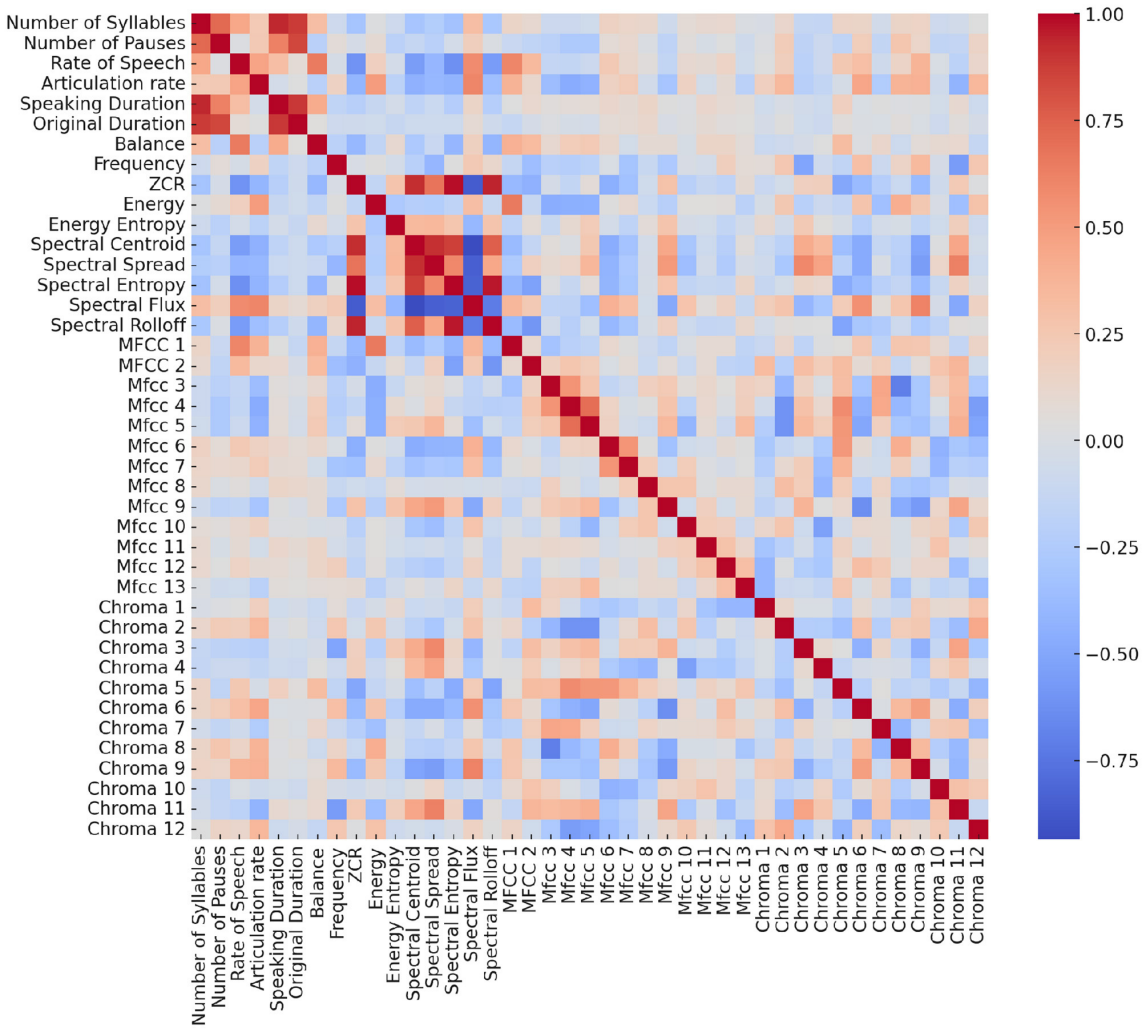
Heatmap of Pearson correlation coefficients among all extracted speech features. The color scale represents the strength and direction of correlations (red = strong positive, blue = strong negative).

To explore the relationships between these features, we calculated Pearson correlation coefficients, measuring the degree and direction of linear relationships (see Figure 2). This approach is crucial for identifying redundancies, interdependencies, and unique contributions of each feature, which can enhance model interpretability and performance by mitigating multicollinearity. Several notable patterns emerge:

- **High Correlation Among Rate-Based Features:** The rate of speech and articulation rate are strongly correlated, confirming that faster speech naturally leads to a greater number of syllables articulated per unit time. This redundancy suggests that only one of these features may be necessary for robust classification.
- **Duration and Pause-Related Measures:** Speaking duration, original duration, and balance also show moderate-to-strong correlations, reflecting the intertwined nature of fluency, pause frequency, and overall timing. Longer utterances often correspond with proportionally longer pauses, which are captured in the balance measure.
- **Spectral and Prosodic Overlap:** Several spectral features (e.g., spectral spread, centroid, and flux) cluster together, indicating they capture related aspects of energy distribution and spectral sharpness. This suggests potential dimensionality reduction opportunities for spectral descriptors.
- **Zero Crossing Rate (ZCR):** Notably, ZCR exhibits a relatively high correlation with spectral flux and spectral centroid. This indicates that temporal fluctuations in signal polarity are linked to changes in frequency distribution and energy transitions. Since ZCR is a simple yet computationally inexpensive measure, its strong correlation with more complex spectral descriptors suggests it may serve as a lightweight proxy for certain spectral dynamics in ASD-related speech analysis.
- **MFCC and Chroma Clusters:** MFCCs are highly intercorrelated, as expected given their derivation from the same cepstral representation. Similarly, the 12 Chroma features show block-wise correlations, particularly between adjacent chroma bands, reflecting harmonic relationships inherent in speech tonality.

These findings highlight redundancy across certain features (e.g., rate measures, MFCCs, Chroma coefficients) as well as unique contributions (e.g., ZCR, spectral spread). This informed our decision to test both a full 40-feature set and a reduced 15-feature set, ensuring that classification models are not unduly biased by collinear predictors.

### 3.3 Classification and analysis of ASD using speech features

In this study, two distinct feature sets were used for classification: (1) all 40 features (including MFCCs and Chroma), and (2) 15 selected features after excluding MFCCs and Chroma. It allows us to assess the necessity of spectral features in ASD detection, especially for cases where computational simplicity is prioritized.

- Results with all 40 features: Table 5 summarizes model performances when using all 40 features. Notably, SVM outperformed other models, achieving the highest F1-score of 84.49%, respectively, underscoring its robustness in capturing nuanced ASD-related speech patterns across a comprehensive feature set.

**Table 5:** Comprehensive speech features extracted for analyzing ASD based on 40 features (K = 5).

**Model**	**Accuracy**	**Precision**	**Recall**	**F1-Score**
SVM	0.8360 ± 0.1334	**0.9039** **±** **0.0788**	0.7974 ± 0.1523	**0.8449** **±** **0.1192**
Random Forest	**0.8505** **±** **0.0899**	0.8733 ± 0.0481	0.8215 ± 0.1190	0.8423 ± 0.0724
AdaBoost	0.8253 ± 0.0815	0.8197 ± 0.0904	0.8190 ± 0.1100	0.8153 ± 0.0842
Naive Bayes	0.7776 ± 0.0906	0.7542 ± 0.0833	0.7800 ± 0.1216	0.7630 ± 0.0885
KNN	0.8349 ± 0.0912	0.8153 ± 0.0411	0.8202 ± 0.1232	0.8146 ± 0.0752
Gradient Boosting	0.8415 ± 0.0751	0.8296 ± 0.0741	**0.8318** **±** **0.1094**	0.8267 ± 0.0750
Voting Ensemble	0.8503 ± 0.0908	0.8718 ± 0.0538	0.8272 ± 0.1264	0.8442 ± 0.0782

Bold values indicate the best mean performance within each column; ties are all shown in bold.

To further justify the choice of 5-fold cross-validation, we directly compared it with 10-fold GroupKFold using the same 40-feature set. As shown in Tables 5, 6, the 5-fold setting yielded slightly higher mean scores in accuracy and F1 score, while also producing consistently smaller standard deviations across nearly all metrics. In contrast, the 10-fold setting led to greater variability, particularly in recall and F1 score, where the standard deviations were substantially larger. This instability is likely due to the smaller test partitions in 10-fold CV, which magnify the impact of sample heterogeneity given our limited dataset size. Taken together, these results indicate that 5-fold CV provides a more stable and reliable estimate of generalization performance in this study, whereas 10-fold CV introduced higher variance and less consistent outcomes.

- Results with Selected 15 Features (Excluding MFCCs and Chroma): Table 7 shows model performances when MFCCs and Chroma features were excluded, resulting in a reduced 15-feature set. The SVM model performed best under this configuration, achieving an accuracy of 85.77% and an F1-score of 86.27%. These results reveal that while spectral features contribute to model accuracy, a simpler feature set without MFCCs and Chroma can still provide competitive performance, making it a viable option for scenarios prioritizing computational efficiency.

**Table 6:** Comprehensive speech features extracted for analyzing ASD based on 40 features (K = 10).

**Model**	**Accuracy**	**Precision**	**Recall**	**F1-Score**
SVM	0.8313 ± 0.1293	**0.8993** **±** **0.0782**	0.7574 ± 0.1940	**0.8123** **±** **0.1439**
Random Forest	**0.8480** **±** **0.1404**	0.8194 ± 0.2104	0.7843 ± 0.2151	0.7834 ± 0.1936
AdaBoost	0.8415 ± 0.1443	0.7021 ± 0.2568	0.7882 ± 0.2252	0.7309 ± 0.2291
Naive Bayes	0.8058 ± 0.1610	0.6806 ± 0.2494	0.7511 ± 0.2338	0.7064 ± 0.2317
KNN	0.8146 ± 0.1440	0.6760 ± 0.2293	0.7692 ± 0.2060	0.7071 ± 0.2007
Gradient Boosting	0.8446 ± 0.1369	0.7279 ± 0.2541	**0.7912** **±** **0.2262**	0.7396 ± 0.2191
Voting Ensemble	0.8404 ± 0.1434	0.7698 ± 0.2489	0.7825 ± 0.2191	0.7629 ± 0.2222

Bold values indicate the best mean performance within each column; ties are all shown in bold.

**Table 7:** Comprehensive speech features extracted for analyzing ASD without MfCC and chroma.

**Model**	**Accuracy**	**Precision**	**Recall**	**F1-Score**
SVM	**0.8577** **±** **0.1133**	**0.9128** **±** **0.0701**	**0.8213** **±** **0.1351**	**0.8627** **±** **0.1052**
Random Forest	0.8447 ± 0.1078	0.8579 ± 0.0632	0.8195 ± 0.1390	0.8354 ± 0.0987
AdaBoost	0.8308 ± 0.1131	0.8217 ± 0.0958	0.8109 ± 0.1476	0.8138 ± 0.1150
KNN	0.7993 ± 0.1111	0.7612 ± 0.0882	0.7885 ± 0.1502	0.7715 ± 0.1096
Gradient Boosting	0.7833 ± 0.0924	0.7553 ± 0.0706	0.7785 ± 0.1288	0.7624 ± 0.0850
Naive Bayes	0.7627 ± 0.0914	0.7084 ± 0.0328	0.7604 ± 0.1374	0.7297 ± 0.0776
Voting Ensemble	0.8482 ± 0.1179	0.8683 ± 0.0898	0.8209 ± 0.1460	0.8424 ± 0.1183

Bold values indicate the best mean performance within each column; ties are all shown in bold.

### 3.4 Analysis of feature importance

Feature importance analysis was conducted to determine which speech features are most indicative of ASD. The top features were identified based on Mean Decrease in Impurity (MDI) scores from Gradient Boosting for the 40-feature set and permutation importance for SVM in the reduced feature set.

**Figure 3:**
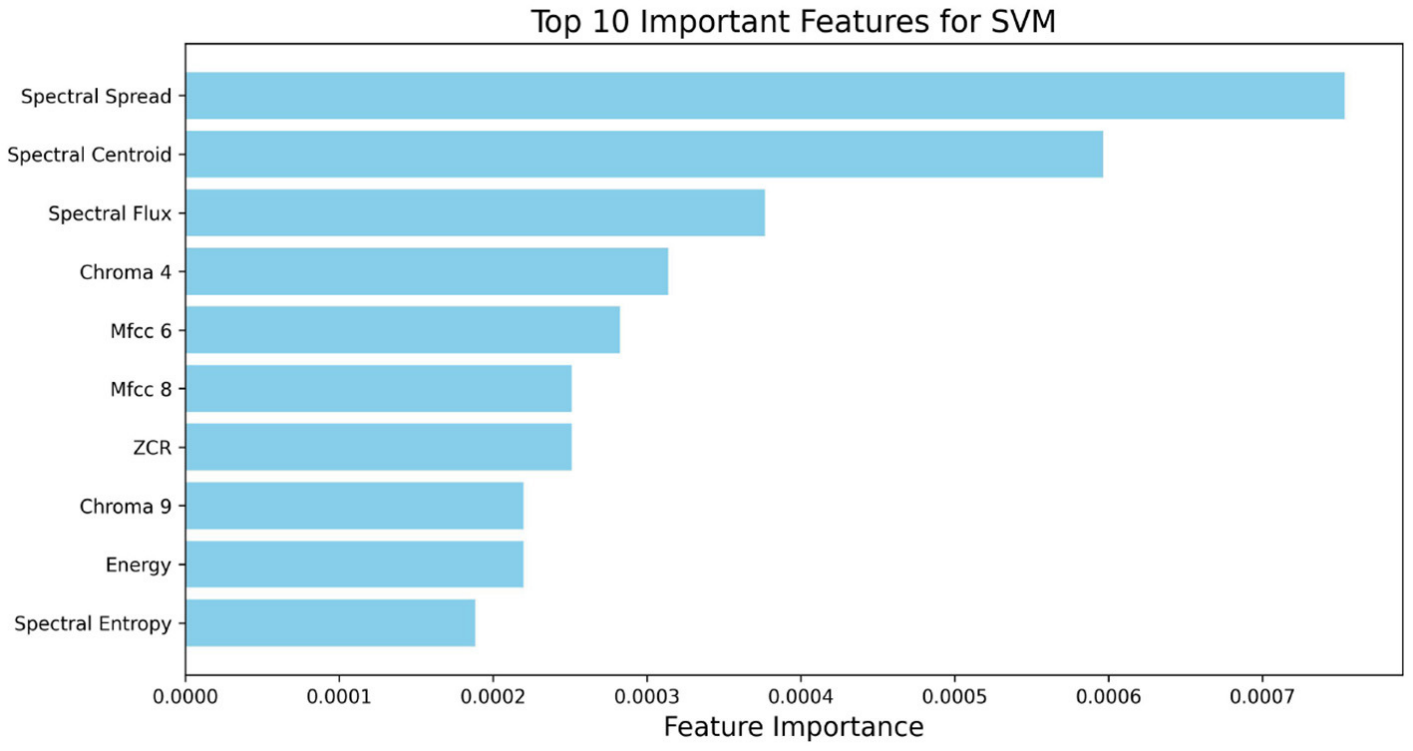
Top 10 important features based on full 40-feature set for ASD classification based on SVM.

To understand the contributions of each feature in ASD classification, we analyzed feature importance using the SVM model with all 40 features. Figure 3 shows the top 10 most important features.

In the analysis with the full 40-feature set (as shown in Figure 3), Spectral Spread and Spectral Centroid were the top features, underscoring the importance of spectral distribution in identifying ASD-related speech abnormalities. Spectral Flux and Chroma 4 also contributed significantly, indicating that both spectral energy distribution and pitch variation are relevant for SVM-based classification. The high importance of MFCC 6 for both models highlights its role in capturing timbral aspects of speech that are characteristic of ASD.

**Figure 4:**
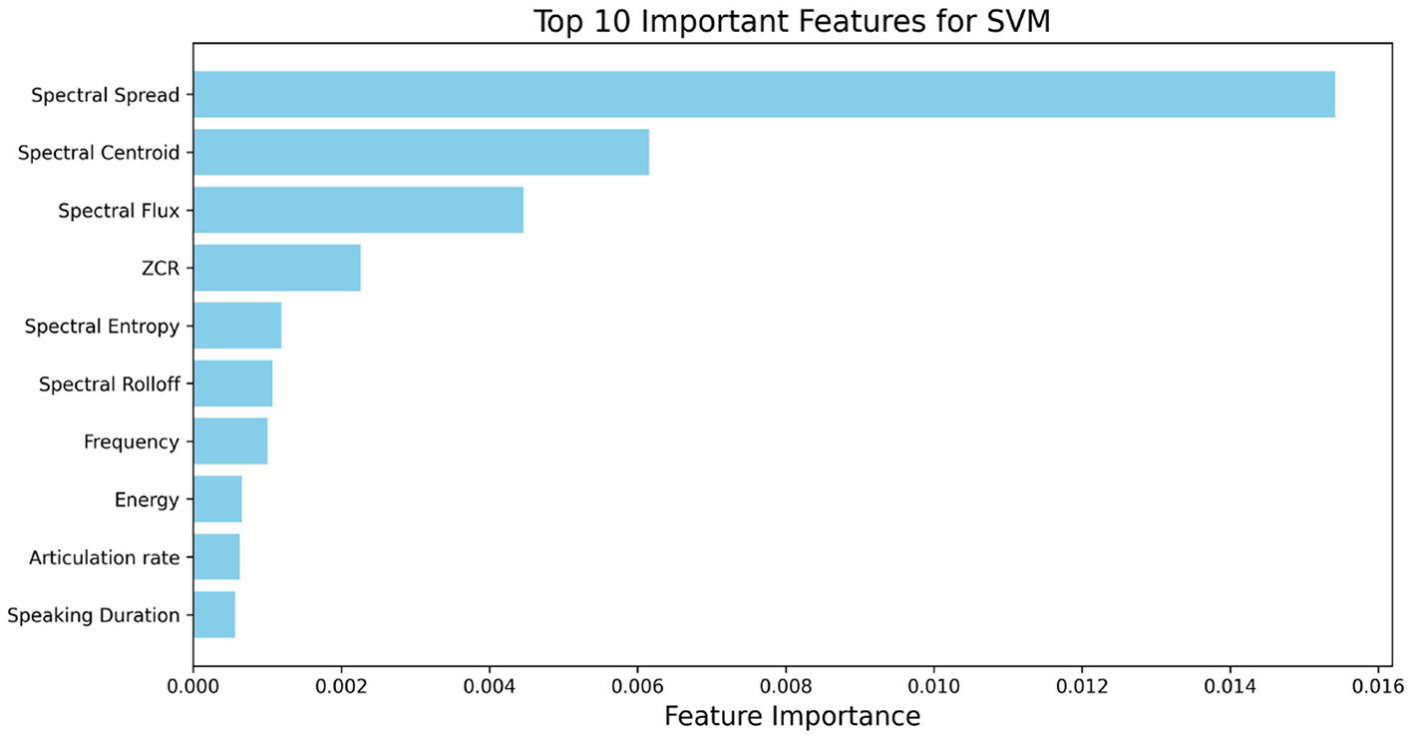
Top 10 important features based on 15-feature set for ASD classification based on SVM.

With this reduced set (Figure 4), Spectral Spread shows by far the largest average contribution, followed by Spectral Centroid. Spectral Flux also ranks highly, with ZCR contributing to a moderate degree. These results suggest that variation in spectral energy distribution (spread, centroid, flux) constitutes the most informative set of cues for classifying ADOS-2 A2 speech abnormalities in this cohort, with additional contributions from temporal zero-crossing and entropy-based measures.

**Figure 5:**
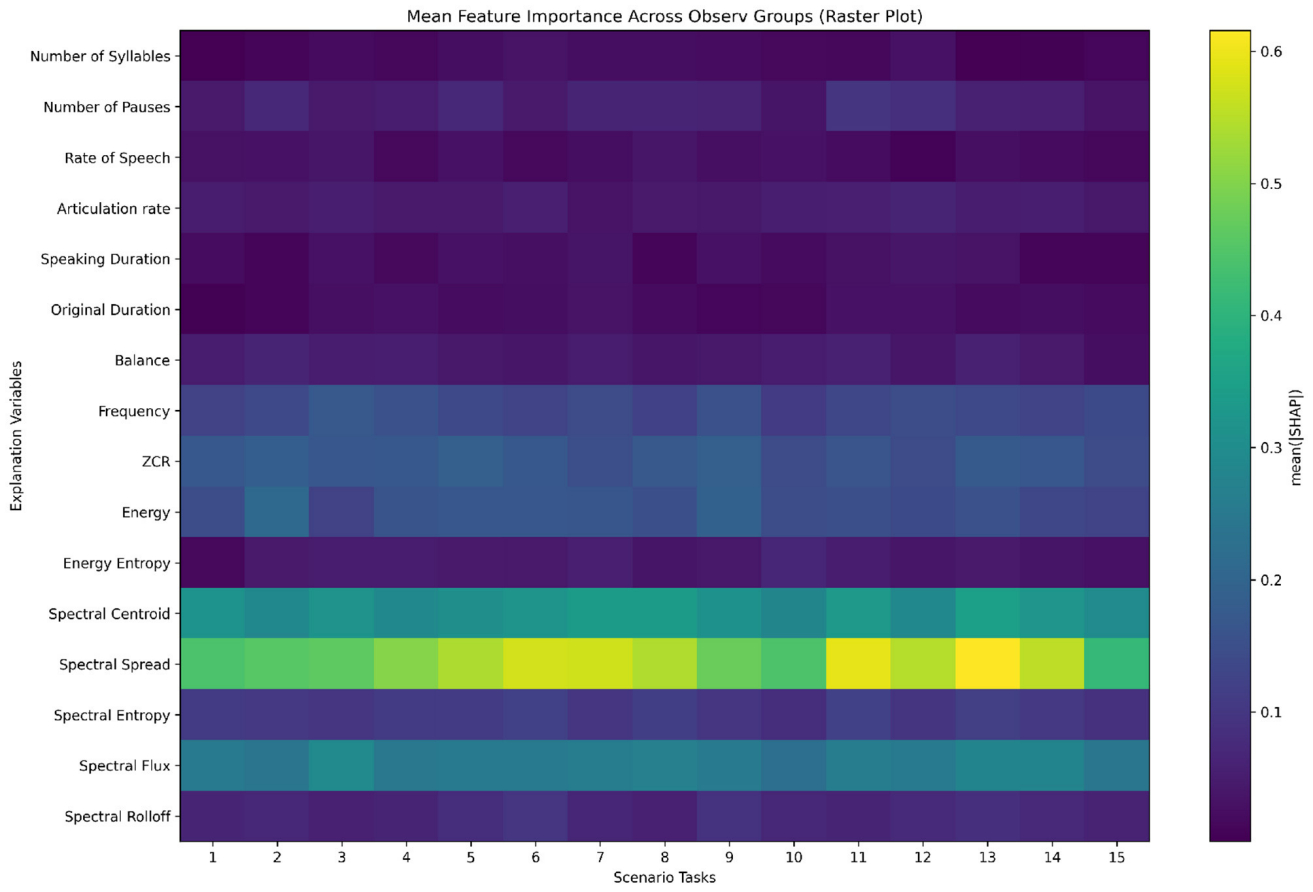
Mean feature importance across 15 scenario tasks in ADOS interviews for diagnosis of speech abnormalities in ASD.

For the scenario-based analysis, we restricted attention to the reduced set of 15 features. This choice was made because (1) the 15-feature set achieved comparable or better performance than the full 40-feature set, and (2) many excluded features (e.g., MFCC, Chroma) are difficult to interpret in clinical or linguistic terms. By focusing on interpretable prosodic and energy-related features, the scenario-level analysis provides insights that are both stable and meaningful for understanding ASD-related speech abnormalities. Figure 5 shows the importance of the 15 selected features across the 15 standardized ADOS scenario tasks, highlighting how feature relevance varies with interactional context and enhancing model interpretability.

From Figure 5, several key patterns emerge. For instance, spectral spread and spectral centroid consistently exhibit relatively high importance across most scenarios, indicating their stability and universal significance in diagnosis of speech abnormalities in ASD across different contexts. Additionally, in Scenario Task 11 and Scenario 13, spectral spread shows particularly high importance, suggesting that these features may capture critical ASD-related speech patterns specific to that task.

## 4 Discussion

This study demonstrates that analyzing specific speech patterns in examiner-patient dialogues can significantly aid in diagnosing ASD. By focusing on a comprehensive set of 40 speech-related features, we examined the roles of intonation, volume, rate, pauses, spectral characteristics, Chroma, and duration in distinguishing individuals with ASD. Our findings suggest that a targeted subset of these features–primarily prosodic and non-spectral characteristics–may offer more effective and computationally efficient diagnostic tools. This discussion addresses the implications of these results, their alignment with existing research, study limitations, and potential directions for future work.

### 4.1 Interpretation of key findings

Our results showed that, while the full feature set achieved strong classification performance, removing MFCC and Chroma features led to an improvement in both accuracy and F1-score. The refined model, focusing on prosodic, rhythmic, and selective spectral features, achieved an accuracy of 85.77% and an F1-score of 86.27%, highlighting the diagnostic potential of simpler, non-spectral features in ASD detection. This improvement underscores the relevance of temporal and prosodic features, such as rate of speech, speaking duration, spectral spread, and frequency, which consistently ranked highly in importance. Notably, spectral spread and frequency emerged as top contributors, supporting the notion that abnormalities in speech fluency, rhythm, and energy distribution are pivotal in ASD-related speech analysis.

The inclusion of the Voting Ensemble further demonstrated that combining multiple classifiers can yield more stable predictions compared to relying on a single model. While SVM achieved the highest mean accuracy and F1-score, its estimates exhibited greater fold-to-fold variability. In contrast, the Voting Ensemble offered a favorable trade-off between accuracy and stability, indicating its potential utility in practical applications where robustness is critical. This highlights the importance of ensemble-based approaches in complementing individual classifiers for speech abnormality detection in ASD.

### 4.2 Comparison with previous research

Our approach aligns with existing research that emphasizes the role of prosodic features in identifying ASD-related speech patterns. Previous studies have highlighted irregularities in speech rate, pauses, and intonation as indicative of ASD (McCann and Peppé, 2003; Bone et al., 2015; Holbrook and Israelsen, 2020). However, our findings extend this by quantitatively demonstrating that reducing reliance on MFCC and Chroma features–commonly used in general speech analysis–can enhance ASD-specific diagnostic performance. This contrasts with studies that focus heavily on spectral features alone and suggests that a shift toward prosody-based diagnostics may offer a more targeted approach to capturing ASD-related anomalies in speech.

### 4.3 Implications for clinical practice

These findings can assist in the assessment of speech abnormalities in verbally fluent individuals with ASD. While not intended as a stand-alone diagnostic system for ASD, our approach may complement existing clinical practices by providing objective, data-driven measures of prosody and rhythm abnormalities.

Although spectral features alone have achieved high accuracy in prior studies (Briend et al., 2023), our results highlight that non-spectral features also capture clinically interpretable aspects of prosody and rhythm that are directly relevant to the ADOS-2 A2 assessment. Rather than replacing spectral features, non-spectral features offer complementary value by improving interpretability and aligning closely with clinical constructs.

Furthermore, the identified importance of features tied to ADOS-2 Module 4, specifically the A2 score, underscores the potential for automated analyses to complement clinical assessment by providing objective, data-driven measures of speech abnormalities. This aligns with recent calls for more objective, data-driven approaches in diagnosis of speech abnormalities in ASD to mitigate subjectivity in clinical practice (Zhang and Li, 2024).

Our scenario-based feature importance analysis (Figure 5, Table 2) demonstrates that the diagnostic contribution of speech features is not uniform across tasks. Spectral-domain measures, particularly *Spectral Spread*, consistently emerge as more influential than prosodic timing variables, but their relevance fluctuates depending on the interactional context. For instance, heightened importance of spectral features in scenarios such as S11 (*Daily Living*) and S13 (*Loneliness*) suggests that tasks prompting extended, personally framed, or socially complex responses may accentuate acoustic variability. These context-sensitive effects highlight the value of considering task demands when interpreting speech abnormalities in ASD, and they point toward the development of context-aware diagnostic models.

### 4.4 Limitations and future work

Despite promising results, this study has several limitations. First, the dataset's size and demographic characteristics may limit generalizability, as it was based on specific examiner-patient interactions within the ADOS-2 framework. Further studies with larger, more diverse samples are necessary to validate the findings across different populations and settings. Additionally, while this study focused on specific speech features, there may be other relevant variables, such as linguistic content and contextual information, which could enhance diagnostic accuracy if integrated with the current model.

Building on this study, future research could explore integrating additional multimodal data sources, such as facial expressions, gestures, and gaze, which may complement speech patterns in ASD diagnosis. Such a multimodal approach could provide a more holistic view of communicative behaviors associated with ASD, potentially enhancing the accuracy and robustness of diagnostic models.

Another limitation is the gender imbalance in our dataset (26 male vs. 7 female participants). This reflects the higher reported prevalence of ASD in males compared to females, which is consistent with prior epidemiological findings. However, the small number of female participants limits the ability to draw strong conclusions about whether the observed speech-related patterns generalize across genders. It is possible that prosodic and spectral features related to ASD manifest differently in female participants, an aspect that our current dataset is underpowered to investigate. Future research with more balanced cohorts will be essential to examine potential gender-specific differences in ASD-related speech characteristics and to improve the generalizability of diagnostic models.

In addition, another limitation relates to repeated ADOS-2 sessions in a subset of participants. Approximately 20% of the recordings came from follow-up sessions conducted about six months apart with the same individuals. While these sessions captured different conversational content and thus provided valuable within-subject variability, they also introduced potential non-independence of samples. We did not explicitly model or control for this in the present analysis, which may have influenced the stability of the classification results. Future research should address this by using larger independent cohorts or by applying statistical approaches such as mixed-effects modeling to account for repeated measures.

Another limitation concerns our feature reduction strategy. We focused on a theoretically motivated subset of 15 features, excluding MFCC and Chroma coefficients because of their limited interpretability in the context of ASD-related speech abnormalities. While this choice resulted in slightly improved model performance, it was not a fully data-driven reduction. Future studies could incorporate systematic feature selection methods (e.g., recursive feature elimination, LASSO regularization, or correlation-based filtering) to more rigorously identify and remove uninformative features from the full set of 40 features, potentially leading to further performance gains.

Another limitation concerns the relatively large standard deviations observed in some models (e.g., SVM), which reflect variability across cross-validation folds. This variability likely stems from the modest dataset size and the heterogeneity of speech samples across participants. As a result, model performance may be sensitive to how training and test sets are partitioned. Future research with larger and more balanced datasets will be crucial for improving the stability and generalizability of the models.

Moreover, as the study found variations in feature importance across different scenario tasks, developing context-sensitive models could yield further improvements. By tailoring feature weighting or selection to specific social interaction scenarios, future models could better capture the nuanced ways in which ASD manifests across diverse contexts. Additionally, exploring reinforcement learning or other adaptive learning techniques could help create models that dynamically adjust to individual differences in ASD presentations.

Another limitation is that our dataset included only individuals assessed with ADOS-2 Module 4, which is restricted to verbally fluent participants. Consequently, the results may not generalize to minimally verbal or non-verbal autistic individuals. Future research should extend this approach to other ADOS modules to capture a broader range of the autism spectrum. Additionally, the participant age range (16-37 years) spans adolescence and early adulthood, which may include individuals undergoing vocal maturation. We did not explicitly control for or analyze the potential impact of pubertal voice changes on extracted speech features. As a result, vocal maturation could have introduced additional variability in the data, which should be examined in future research with larger and more age-stratified samples.

In conclusion, this study underscores the potential of prosody-based and scenario-sensitive approaches in diagnosis of speech abnormalities in ASD. By reducing reliance on spectral features and leveraging context-specific analysis, future diagnostic tools may become more precise and accessible, supporting earlier and more objective ASD assessments.

## 5 Conclusion

This study demonstrates that analyzing a targeted set of speech features, particularly prosodic and non-spectral characteristics, can effectively support diagnosis of speech abnormalities in ASD. By examining 40 distinct speech features from examiner-patient dialogues, we identified a reduced feature set focused on prosodic and rhythmic attributes, achieving strong diagnostic accuracy and outperforming models that rely on more complex spectral features. The identified features, such as spectral spread, Spectral Centroid, and Spectral Flux, underscore the relevance of non-spectral cues in capturing ASD-related communication patterns.

These findings suggest that a prosody-focused, streamlined approach can enhance accessibility and efficiency in ASD diagnostics. The performance of the reduced feature set highlights its potential for real-time assessments, supporting quicker and more objective screening for speech abnormalities in ASD. Moving forward, integrating context-sensitive models and multimodal data sources could refine and advance ASD diagnostics, ultimately contributing to improved intervention strategies for individuals on the autism spectrum.

## Data Availability

Publicly available datasets were analyzed in this study. This data can be found here: https://github.com/cbhu523/speech_ASD.
